# Job vacancies at ECDC

**DOI:** 10.2807/1560-7917.ES.2022.27.41.221013v

**Published:** 2022-10-13

**Authors:** Eurosurveillance editorial team

**Affiliations:** 1 European Centre for Disease Prevention and Control (ECDC), Stockholm, Sweden

The European Centre for Disease Prevention and Control (ECDC) invites applications for the following positions:

 

- **Expert Communicable Diseases Prevention and Control **(six posts)
  The application deadline expires on 8 November 2022.

 

For more information, please visit the ECDC website: 

https://erecruitment.ecdc.europa.eu/?page=advertisement

